# Neuroprotection and Neurodegeneration in Alzheimer's Disease: Role of Cardiovascular Disease Risk Factors, Implications for Dementia Rates, and Prevention with Aerobic Exercise in African Americans

**DOI:** 10.1155/2012/568382

**Published:** 2012-04-17

**Authors:** Thomas O. Obisesan, Richard F. Gillum, Stephanie Johnson, Nisser Umar, Deborah Williams, Vernon Bond, John Kwagyan

**Affiliations:** ^1^Division of Geriatrics, Department of Medicine, Howard University Hospital, 2041 Georgia Avenue, NW, Washington, DC 20059, USA; ^2^Division of Cardiology, Department of Medicine, Howard University Hospital, 2041 Georgia Avenue, NW, Washington, DC 20059, USA; ^3^Department of Health and Human Performance, Howard University Hospital, 2041 Georgia Avenue, NW, Washington, DC 20059, USA; ^4^Howard University Hospital, Georgetown-Howard Universities Center for Clinical and Translational Science, 2041 Georgia Avenue, NW, Washington, DC 20059, USA

## Abstract

Prevalence of Alzheimer's disease (AD) will reach epidemic proportions in the United States and worldwide in the coming decades, and with substantially higher rates in African Americans (AAs) than in Whites. Older age, family history, low levels of education, and *ɛ*4 allele of the apolipoprotein E (APOE) gene are recognized risk factors for the neurodegeneration in AD and related disorders. In AAs, the contributions of APOE gene to AD risk continue to engender a considerable debate. In addition to the established role of cardiovascular disease (CVD) risk in vascular dementia, it is now believed that CVD risk and its endophenotype may directly comediate AD phenotype. Given the pleiotropic effects of APOE on CVD and AD risks, the higher rates of CVD risks in AAs than in Whites, it is likely that CVD risks contribute to the disproportionately higher rates of AD in AAs. Though the advantageous effects of aerobic exercise on cognition is increasingly recognized, this evidence is hardly definitive, and data on AAs is lacking. In this paper, we will discuss the roles of CVD risk factors in the development of AD and related dementias, the susceptibility of these risk factors to physiologic adaptation, and fitness-related improvements in cognitive function. Its relevance to AD prevention in AAs is emphasized.

## 1. Introduction

Although anticholinesterase therapies have greatly improved symptomatic treatment of AD, they have not been demonstrated to significantly slow disease progression. Excess morbidity and mortality from AD continue to generate enormous economic burden on families and on the United States. Preservation of intellectual dexterity among those showing earliest symptoms of AD may ameliorate the physical, emotional, and economic burden associated with the disease, and that is an important public health goal.

**Figure 1:**
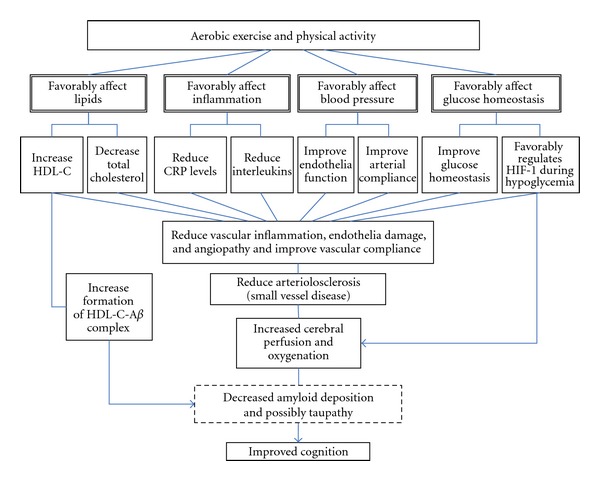
Aerobic exercise training and cognitive functions. Aerobic exercise increases HDL-C and subfractions; decrease total cholesterol, C-reactive protein, and interleukin-1; improves endothelia function and arterial compliance; improves glucose homeostasis and downregulates hypoxia.

A promising evidence-based and relatively side-effect free lifestyle approach is emerging as an alternative or adjunct to anticholinesterase therapy. Specifically, aerobic exercise training has been demonstrated to improve cognitive function (Figure 1). Though, the effect sizes for these studies were surprisingly large, and the results fairly consistent, however, the sample sizes were small and included mostly Whites. Importantly, the mechanism by which an advantageous effect occurs is yet to be systematically examined. Remarkably, aerobic fitness can improve many of the putative AD risk factors such as high-density lipoprotein cholesterol (HDL-C), inflammation, and arteriolosclerosis. However, improvements in these risk factors have not been optimally explored as potential mechanisms by which aerobic training improves cognitive function in humans and AA in particular. Given that AAs (i) have higher incidence and prevalence of AD than Whites, (ii) have paucity of cross-sectional and lack prospective data on the beneficial effect of exercise on cognitive function and (iii) are more sedentary relative to Whites, in whom data show the beneficial effect of exercise, and therefore have more room for exercise-induced improvements in risk, it is relevant that the beneficial effects of aerobic fitness on neurocognitive processes is prospectively examined in this population.

## 2. Magnitude of Alzheimer's Disease Burden

Clinically, AD is a constellation of gradual decline in memory, other cognitive functions, behaviors, and activities of daily living leading to total dependency [1]. Pathologically, AD is a heterogeneous neurodegenerative disorder characterized by amyloid-beta plaques (A*β*), neurofibrillary tangles, inflammation, and neuronal loss. AD is the most common type of dementia constituting ~2/3rd of all late-life dementias and is estimated to affect 8 percent of persons age 65 years or older [2]. The prevalence of AD increased about 15-fold from 3 percent among individuals between the ages of 65, and 74 years to 47 percent for persons age 85 and older [3]. Also, the incidence of AD increased from 0.5 percent per year at age 65, to ~8 percent per year over age 85 [4, 5]. Without AD, hypertension, and other chronic age-related medical conditions, many older persons would remain relatively functional until late in life, contributing to society. That would reduce the nation's dependency ratio [6]. Based on 1999 estimates, the annual health care cost for AD was ~$100 billion [7]. Excluding ~$202 billion in uncompensated care by ~15 million families and caregivers, total payments in 2011 for health care, long-term care, and hospice services for people aged ≥65 years with AD and other dementias were estimated to be $183 billion [8]. Given this staggering cost and the projected increase in elderly population by the year 2050, identifying effective mechanisms to ward off structural and functional declines of AD is an important public health goal.

## 3. AD in African Americans

The incidence and prevalence of AD is higher in AAs than Blacks from Sub-Sahara African and compared to persons of European descent. In spite of this statistics, the disease is understudied in AAs [9]. In the Multi-Institutional Research on Alzheimer's Disease Genetic Epidemiology (MIRAGE) study led by Farrer et al., the adjusted cumulative risk of dementia in the first degree relatives of probands with AD in AAs was approximately twice that of a similar White sample. According to reports from the Indianapolis-Ibadan Dementia Project, the rates of AD and dementia in Yoruba (an ancestral population in Nigeria) are less than half the rates in AAs [10], suggesting possible contributions from the environment.

To better discern the relatively high rates of AD in AAs, a number of studies have compared the prevalence and incidence of AD and related disorders across populations in the US. Whereas a faster rate of cognitive decline in Mild Cognitively Impaired (MCI) AAs than in non-AA was observed in one study that used a community-based sample [11], others found no evidence of racial disparities in trajectories of MCI [12, 13]. However, in AAs compared to Whites, a significantly slower rate of cognitive decline was reported once AD begins [13, 14]. For example, using age and education adjusted growth curve approach to estimate individual paths of change in global cognition, Barnes reported that older AAs had a lower level of global cognition at baseline and declined at ~25% slower rate compared to Whites [14]. In another study that examined the severity of AD at the time of presentation to the medical establishment among different ethnic groups in the US, minority persons (including AAs) compared to Whites tended to exhibit a more severe profile of AD at the time of presentation [15]. Despite such relatively slower rate of AD progression, AA MCI and incident AD patents experienced greater decline in body mass index (BMI) compared to normal controls [16]. While the biologic explanation for the lower rates of cognitive decline in AAs needs further elucidation, an enriched social network has been proposed as a possible explanation [17]. Collectively; a higher incidence and greater rate of cognitive decline in MCI and AD-afflicted AAs, delayed diagnosis, lower rate of cognitive decline once AD occurs together with an accelerated weight loss suggest that the overall prevalence of AD in this population will reach epidemic proportions in the coming decades. Decreased overall wellness and increased health disparity are notable consequences. Given the effects of socioeconomic variables and access to health care on these important health indicators, such consequences may become blurred by regional variations. In support of this view, we recently reported that racial differences in AD or dementia mortality varied by regions in the United States (Table 1) [12, 18]. A fundamentally important implication of these observations is that other factors at the environment or genetic level may contribute to higher incidence and prevalence of AD in AAs than in Whites. Increased CVD risks and low levels of physical activity may explain some of these differences.

At the genetic level, APOE gene is the most consistent nondeterministic genetic risk factor for AD. Its contributions to AD risk are graded across alleles (*ɛ*2, *ɛ*3 and *ɛ*4), with *ɛ*4 conferring the highest risk [19, 20]. While many believed that the contributions of the *ɛ*4 allele to AD risk are similar across populations [21, 22], others have reported a lower associated AD risk in AAs than in Whites [23, 24]. Using pooled samples from the MIRAGE, Alzheimer's Disease Neuroimaging Initiatives (ADNI), Canadian Study on Genetics of Alzheimer's Disease Association (GenADA), and National Institute on Aging-Late-Onset Alzheimer's Disease Family Study (NIA-LOAD) data, we reported that the presence of *ɛ*4 allele significantly and exponentially associated with AD in AAs in a dose-dependent manner. However, the odds ratio estimates in *ɛ*4 carriers showed lower rates of AD in AAs compared to Whites (63.1 percent versus 67 percent). Conversely, we also observed a higher occurrence of *ɛ*4 in AA controls than White controls (40.1 percent versus 29.1 percent), respectively [25]. These suggest that the *ɛ*4 allele of the APOE gene may interact with other risk factors to cause a differential AD risk in AAs compared to Whites. Interestingly, AAs also have increased CVD risks such as hypertension, diabetes, and hypercholesterolemia. Evidently, key interactions of APOE gene with CVD risk such as lipids, inflammation, glucose homeostasis, and lifestyle factors in these populations must be considered [26]. Such factors may lend themselves to interventions capable of attenuating AD risk in AAs and other populations at risk.

At the environment level, growing evidence indicates that aerobic fitness can reduce AD risk in predominantly White samples. However, these advantageous effects of exercise are yet to be validated in a relatively more sedentary AA sample [27]. Given the higher rates of AD in AAs than in Whites and the lack of substantive differences in AD neuropathology [28, 29], it is likely that AA mild AD patients will also benefit from the advantageous effects of aerobic fitness. In addition to the public health imperative, such intervention may ameliorate the physical, emotion, and economic burden associated with AD. All of these effects will benefit society at large.

## 4. Rationale for Dementia Prevention

Whereas, it is established that the preservation of neurocognitive function among those showing earliest signs and symptoms of AD can attenuate the burden associated with the disease; unfortunately, this benefit and the national goals of Healthy People 2010 cannot be realized without an efficient AD prevention strategy. Moreover, while medical treatment after disease onset may reduce disease progression and mortality, eventually, increases in disease prevalence will substantially escalate total disease burden and healthcare cost for the population. Though the current approach to symptomatic treatment of AD may not be cost-effective in populations with excessive rates of disease such as AAs, a low-cost low-risk intervention strategy with dual applicability for primary and secondary prevention is likely to be advantageous.

The goal of this paper, therefore, is to enhance scientific discuss on the role of CVD risk in the development of AD and related dementias and to add clarity to the clinical utility of fitness adaptation in preventing AD in those at risk. However, significant uncertainty in disease progression from prodromal to symptomatic AD raises an important question of whether intervention should be directed at the fully characterized MCI or AD clinical phenotypes. Because of the present impracticality of reversing neuronal death underlying the AD phenotype, interventions are likely to yield the most benefit if initiated at the earliest possible stage as in pre-MCI. Indicators of such timely intervention may include notable endophenotype such as decreasing cerebrospinal fluid (CSF) levels of A*β* that precede the emergence of the MCI clinical phenotype. If confirmed in randomized clinical trials, aerobic fitness can become an effective public health tool to combat AD risk. Such a low-cost low-risk effective strategy is likely to reduce the burden of disease and optimize the well-being of older adults at increased AD risk.

## 5. Cardiovascular Disease Risk in AD Development

Stroke and Alzheimer's type dementia increase at comparable rates with advancing age. Atherosclerosis, hypertension, diabetes mellitus, and lipids are major CVD risk factors shown to be associated with AD [30]. Recently, Arvanitakis and colleagues reported an association of diabetes with semantic memory impairment in both Blacks and Whites [30]. The Rotterdam population-based prospective study that examined approximately 8000 subjects over age 55 for the frequency of lifetime risk of dementia and its subtypes, including AD, showed an increase in the prevalence of atherosclerosis in both vascular dementia and AD [31]. Also, compilation of autopsy reports on AD brains indicate, that approximately 60–90 percent of the cases exhibited variable cerebrovascular pathology synonymous with CVD [28–32]. In AD cases ascertained by the presence of amyloid angiopathy, endothelial degeneration, and periventricular white matter lesions at autopsy, Van Nostand showed that ~1/3rd had evidence of cerebral infarction [33]. However, in a study to examine the relationship of important AD intermediate phenotype such as differences in brain volume, hippocampal volume and cerebrovascular risk factors, and APOE4 among MCI subtypes, He and colleagues found CVD risk factors to be more closely related to nonamnestic MCI and vascular dementia; though emphasized that the biological differences between amnestic (AD group) and nonamnestic (presumed vascular etiology) were very subtle [34]. Given these observations, it is possible that CVD risks plays a greater role in cognitive decline in older AAs compared to Whites. In support of this view, Brickman et al. demonstrated more severe white matter hyperintensity (WMH) burden in AAs and Hispanics compared to Whites [35]. In particular, vascular disease was associated with relatively smaller brain volume and higher WMH burden in AAs. Others have also demonstrated greater degree of psychomotor impairment, a surrogate for higher cerebrovascular burden in AAs than in Caucasians [36]. Collectively, these reports indicate that CVD risk factors may also influence cognitive loss, particularly in AAs who suffer a greater burden of CVD risk and related brain pathology.

Regardless of whether increased CVD risk burden culminates into vascular dementia, enables or directly promote AD pathology [37–42], with or without interactions with age-associated decline in health status [43], interventions directed at reducing CVD risk factors may attenuate declining cognitive dexterity especially in older AAs. Despite the evidence showing a higher degree of CVD risks and cerebrovascular pathology in AAs, data is lacking on whether aerobic fitness-induced reduction in CVD risk can concomitantly reduce AD risk in this population. Given that AAs suffer a high CVD-related morbidity, they are likely to benefit from CVD risk reduction measures. Collection of prospective data on putative CVD mediators of AD and their susceptibility to fitness adaptation will elucidate its clinical utility in ameliorating AD risks in AAs and other populations.

## 6. Mechanisms by Which Cardiovascular Disease Risk Can Influence AD

### 6.1. Association of Total Cholesterol with AD Risk

Disorder of brain cholesterol metabolism has been associated with all principal pathological features of AD such as synaptic transmission [44], amyloid [45], and tau pathology [44]. Lipids and lipid peroxidation products have important roles in the homeostasis of the central nervous system [46]. In animal and in vitro studies, Golde and colleagues showed that overexpression of cholesterol resulted in the formation of amyloid *β* and contributed to the degradation of neurons and subsequent cognitive impairment [47]. Also, lipid transport genes and vascular changes associated with peripheral dyslipidemia have been associated with an increased risk of AD. This indicates that lipids may be involved in the pathogenesis of neurodegeneration and related dementias. Alternatively, lack of cholesterol supply to the neurons via lipoprotein transport may cause failure of neurotransmission and synaptic plasticity [48]. However, because almost all brain cholesterol is a product of local synthesis, with brain blood barrier efficiently protecting it from exchange with lipoprotein cholesterol in the systemic circulation [49], serum cholesterol may not accurately reflect the related AD risk. Moreover, the bimodal relationship of serum cholesterol with health may contribute to the inconsistencies of reports on the association of cholesterol with cognitive health, especially when the protective influence of HDL-C is not considered.

### 6.2. Association of High-Density Lipoprotein Cholesterol with AD and CVD Risk

 HDL-C is an important risk factor for CVD [50, 51]. As with CVD risk, the contribution of HDL-C to AD risk is increasingly recognized. HDL-C functions to both keep its lipid components soluble and also provide an efficient mechanism for their transportation through plasma and to or from the tissues. Low HDL-C, together with suboptimal transport system in humans, results in gradual deposition of lipid (especially cholesterol) in tissues causing arteriolar narrowing and chronic cerebral oxygen deprivation [52].

### 6.3. HDL-C Is the Predominant Lipoprotein in Human Brain Circulation, and Its Low Levels Have Been Associated with Impaired Memory [53–55]

**Figure 2:**
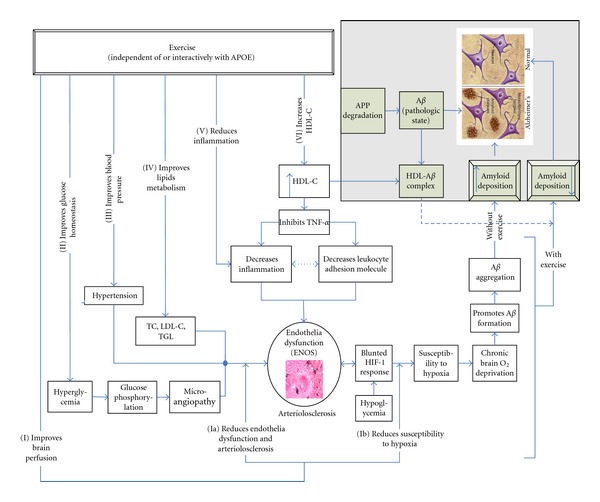
Interaction of HDL-C with AD risk factors. Relationships of exercise to prevention of intracerebral amyloid deposition.

For example, Wolf and colleagues recently showed that low levels of HDL-C and not LDL or total cholesterol levels were associated with hippocampus atrophy in aged humans [56]. Unlike total cholesterol, HDL-C brain level correlates with its plasma concentration. This evidence suggests that low levels of HDL-C may play an important role in AD risk. Beyond the direct effect of low HDL-C on arteriolosclerosis, high HDL-C may conversely influence AD risk in three other important ways: (i) mediation of reduced inflammatory cytokines which is central to arteriolar narrowing; (ii) through its interaction with A*β* to form soluble HDL-C-A*β* complex (Figure 2); (iii) its antioxidant property.

### 6.4. Evidence of Anti-Inflammatory Effects of HDL-C

In support of HDL-C anti-inflammatory effects, Cockerill and colleagues showed that, in physiological concentration, isolated plasma HDL-C inhibited tumor necrosis factor-*α* (TNF-*α*) or interlekin-1 (IL-1) and reduced leukocyte adhesion molecules in a concentration-dependent manner (Figure 2) [57]. Others have reported increased markers of inflammation with low HDL-C levels [58]. Therefore, as the predominant lipoprotein in the brain circulation, the anti-inflammatory effects of high HDL-C may play an active role in reducing vascular inflammation and arteriolosclerosis of the cerebral circulations. This may enhance brain oxygenation and preserve neurocognitive dexterity.

### 6.5. Evidence of Antiamyloid Deposition Effects of HDL-C

The interaction of HDL-C with A*β* is consistent with its neuroprotective effects. For example, HDL-C attenuates the aggregation and polymerization of A*β* protein (Figure 2) [59]. Using thioflavin T fluorescence, Olesen and Dagø showed that HDL-C reduced amyloid formation in vitro. Additionally, the association of HDL-C with A*β* was also recently demonstrated by Koudinov et al. who isolated HDL-A*β* complexes from CSF [60]. More support for the direct effects of HDL-C on A*β* was evidenced by studies showing that A*β* mediated the cellular uptake of lipoproteins [61], and that HDL-C induced increases in the cellular degradation of A*β* in cultured microglia [62]. Its neuroprotective property against A*β* was also demonstrated by Farhangrazi et al., who showed that the neurotoxic effect of A*β* in cortical cell cultures became attenuated in the presence of high levels of HDL-C [63]. It is therefore likely that HDL-C exerts a significant antiamyloid effect that may be susceptible to lifestyle alteration.

### 6.6. Evidence of Antioxidant Effect of HDL-C

Growing evidence suggests that oxidative damage is implicated in neuronal degeneration that occurs in AD brains [64, 65] High-plasma HDL-C particles can also exert antioxidant activity and have the capacity to protect low-density lipoprotein (LDL) against oxidative stress [66]. Though the exact mechanism by which HDL-C exerts antioxidant effects needs further clarification, its role as a transporter of enzymes exerting antioxidative activity such as paraoxonase (PON) [67], platelet-activating factor acetylhydrolase (PAF-AH) [68] and lecithin-cholesterol acyltransferase (LCAT) [69] must be noted. Moreover, intrinsic antioxidative property of HDL-C subfraction is deficient in the presence of low HDL-C phenotype and amplified by low number of circulating HDL-C particles. Indeed, this dysfunctionality is closely related to elevated oxidative stress evidenced by breakdown products of arachidonic acid such as plasma isoprostane.

In summary, given the effects of high HDL-C levels on the biochemical properties of A*β* and its antioxidant property, it is likely that HDL-C plays a direct role in brain amyloid deposition and AD risk. Because high HDL-C can reduce inflammation, enhance lipid metabolism, and therefore reduce arteriolosclerosis and enhance brain perfusion, it is likely to be important for optimal neurocognitive function. Fortunately, HDL-C is susceptible to the effects of aerobic exercise training. Our own aerobic fitness data indicated that a 6-month aerobic exercise-training can improve protective HDL-C large particle size in AAs [70]. Whether this improvement translates into improvement in cognitive function is the subject of our ongoing investigations.

### 6.7. Association of Inflammation with AD Risk

Though considerable uncertainty exists on the exact role of the inflammation in AD, many studies have documented the association of inflammatory markers such as CRP and IL1a with AD. The role of inflammation has become even more evident with recent studies on microglia. Microglia, a distinct population of brain-resident macrophages, is indicative of ongoing chronic inflammation in AD. In support of anti-inflammatory role of microglia, Minagar and McGeer demonstrated its activation in regions of the brain showing AD pathology [71, 72]. Building on earlier observations, Frank et al. recently examined the association of inflammation with the neuropathology of AD and showed that microglia are present in close association with aggregated types of A*β* plaques and around neurofibrillary tangles [73]. Frank et al. also showed that microglia-derived factors including reactive oxygen species and tumor necrosis factor-*α* (TNF-*α*) are neurotoxic [73]. Neuronal damage by microglia can also occur when activated microglia and reactive astrocytes surrounding intracellular deposits of A*β* protein initiate an inflammatory response [74]. Often, this type of response is characterized by local cytokine-mediated acute phase response and activation of the complement cascade [74].

However, studies on the effects of anti-inflammatory agents on AD risk are inconclusive. For example, a retrospective study of long-term users of nonsteroidal anti-inflammatory drugs showed a lower incidence of AD in this population [75]. Conversely, recent clinical trials found no benefit to the use of nonsteroidal anti-inflammatory drugs (NSAIDs) [76, 77]. Since the actual dose, duration, and period of protective NSAID use are unknown, these negative results are hardly definitive. Further, many of these studies did not account for genetic mediators of inflammatory markers. Notable among such markers are interleukins and C-reactive protein (CRP). We and others have shown that aerobic fitness either independently or interactively with its genetic mediators can reduce CRP level [78, 79]. Whether training-induced reductions in inflammation and CRP levels translate into improvements in cognitive performance has not been studied. In our currently ongoing pilot clinical trial, we will further delineate the role of inflammation in AD, its association with HDL-C and A*β* protein, and whether exercise-related changes in inflammatory markers are associated with neurocognitive measures that are used in this study. Demonstration of concomitant reduction in inflammatory markers, with improvements in neurocognitive function after aerobic exercise training, will be important evidence supporting the role of inflammation in AD. This would be indicative of the susceptibility of AD risk factors to aerobic fitness.

## 7. Association of Hypertension with AD Risk

Growing evidence indicates a causal role of hypertension for cognitive decline of the Alzheimer's type dementia (Figure 2) [80, 81]. A few longitudinal studies have also emphasized a connection between high blood pressure in midlife and dementia in late life [80, 81]. Recently, Korf and colleagues reported an association of systolic blood pressure (SBP) and pulse pressure (PP) with medial temporal lobe atrophy (MTA), a hallmark of AD, in individuals with late onset dementia, especially when coexisting with white matter changes [82]. These reports indicate that CVD risk factors including hypertension may also influence AD risk. Our own data from the NHANES III support these observations. Though the optimal BP for cognitive performance remains poorly defined and evidence is emerging on the effects of CVD-related genes such as ENOS and ACE on AD, the combined effects of hypertension and genetics on neurocognitive function need clarity. Like many other CVD-related AD risk, considerable evidence suggests that fitness adaptation can reduce blood pressure [83–85]. However, whether a concomitant improvement in neurocognitive performance occurs with aerobic fitness-related improvements in blood pressure has not been examined. Even if very small cognitive benefit accrues from blood pressure reduction, substantial gains can be realized, given the relatively high prevalence of hypertension in the United States and in the World.

## 8. Association of Hypoxia and Glucose Homeostasis with AD Risk

Neurons are highly vulnerable to impairments of oxygen homeostasis because of their singular dependency on oxygen. Though the human brain averages about 2 percent of body mass, it utilizes 15 percent of cardiac output and 20 percent of respiratory oxygen uptake. In neural cells in primary culture and in the hippocampus using in vivo models, both cycloo-2 (COX2) and presenile-1 (PS1) are induced after only about 5 minutes of hypoxia [86, 87]. Cell cultures and transgenic models also suggest an interactive relationship of hypoxia with microglia activation, neuroinflammation, reduced neuronal function, and apoptosis [88–90]. These reports are indicative of the independent and collective roles of CVD risk factors and, importantly hypoxia in AD risk.

Changes in brain glucose metabolism are associated with AD [91, 92], and the upregulation of glucose metabolism has been demonstrated to activate the transcription of hypoxia inducible factor (Figure 2) (HIF-1) [93]. HIF-1 is a heterodimeric transcription factor comprised of two subunits, HIF-1*α* and HIF-1*β*. In normoxic state, the binding and transcription of hypoxia-inducible genes do not occur [94]. HIF-1 mediates the adaptation of cells to hypoxia and hypoglycemia by upregulating genes involved in glucose transport and glycolysis [93]. Blunted HIF-1 response to hyroxia has been shown to promote A*β* formation and changes in glucose metabolism. Together, this evidence suggests that inflammation, acting in concert with HIF-1, and glucose metabolism may play an active role in brain cellular damage and ultimately AD. Fortunately, fitness adaptation can enhance glucose uptake, increase cerebral perfusion and possibly favorably regulate the activation of HIF-1. However, there is no randomized, controlled experiment linking aerobic fitness to improvements in these intermediate phenotypes or neurocognitive function. Large-scale clinical trials are needed to determine whether fitness-related improvements in brain perfusion are effective intervention strategies to reduce AD risk.

## 9. Exercise Effects on Cognitive Function

### 9.1. Fitness Training Is Associated with Improved Cognitive Health in Cross-Sectional and Few Prospective Studies

Cross-sectional [95, 96], longitudinal [97], and meta-analyses have demonstrated that improvements in cardiovascular fitness can improve cognitive function in humans [98, 99]. For example, Larson recently showed a <3 times/week exercise to be related to increased risk of AD compared to >3 times/week exercise [100]. Others have reported an inverse relationship of AD with the number of physical activities performed. [96] In a study of leisure-time physical activity during midlife and dementia, Rovio et al. reported a reduced risk of AD in those with higher levels of physical activity [95]. These studies suggest a significant association of physical activity with later reduction in neurocognitive function and dementia. Notwithstanding the mostly beneficial effects of exercise observed in the majority of studies, limitations such as self-reported data; failure to distinguish between aerobic and no-aerobic activities; failure to assess exercise duration, intensity, and frequency; differences in the volume of exercise that is beneficial likely resulted in significant variability among studies.

To obviate the limitation inherent in cross-sectional studies, a few prospective studies have examined the effects of fitness adaptation on memory. Using meta-analyses of 18 published studies, Colcombe and Kramer found a beneficial effect of fitness training on an array of neurocognitive process in nondemented older adults [99]. In a ~7-year prospective study of 5925 older women, Yaffe et al. demonstrated a 37% reduction in the odds of cognitive decline in 3rd quartile compared to 1st quartile of physical activity [101]. In another prospective study, Barnes and colleagues reported better cardiorespiratory fitness at baseline to be associated with less cognitive decline at ~6-year followup [102]. A recent 24-week randomized placebo control trial of an unsupervised physical activity intervention study in MCI-like subjects by Lautenschlager et al. revealed an improvement of 1.0 points in ADAS-cog for exercisers, and a deterioration of 1.3 for controls yielded a total of 2.3 point difference between the intervention and control groups over 6 months. Interestingly, the cognitively beneficial effects of aerobic fitness remained at 18-month followup [103]. Though these prospective studies add substantially to the current knowledge and the directionality of the relationship of aerobic fitness with neurocognitive function, data is lacking on AAs. Importantly, a more rigorous randomized controlled trial in MCI patients is needed to establish causality and to clearly delineate the overall volume of exercise that is beneficial. In spite of the skepticism on the relatively large aerobic fitness-related effect size reported in many studies, the multiple levels at which exercise can influence AD risk support such observations.

## 10. Mechanism by Which Exercise Influences Neurocognitive Function

**Figure 3:**
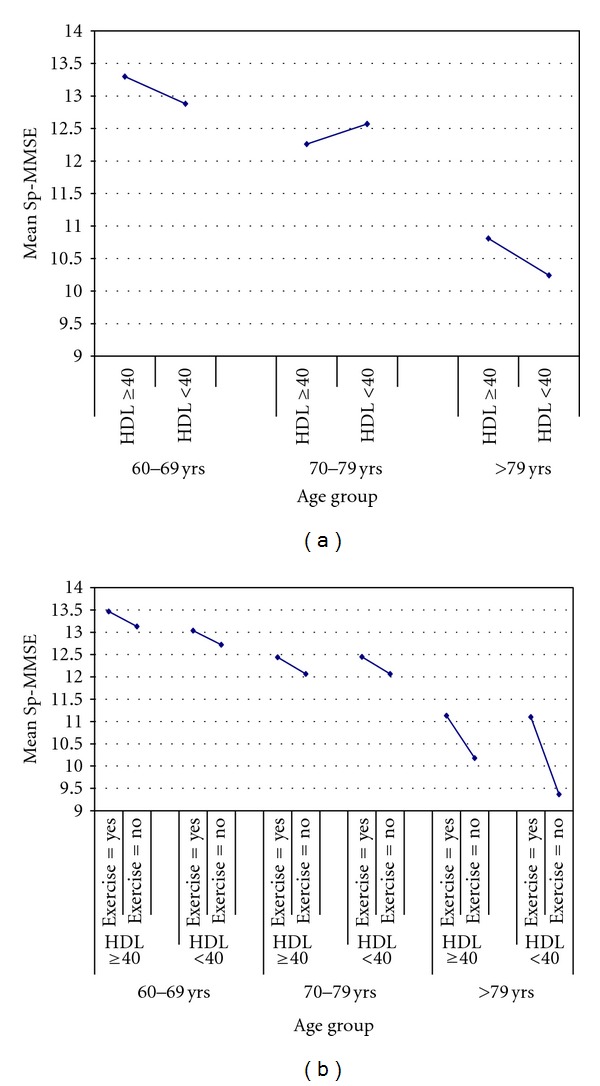
Adjusted mean short portable MMSE by HDL-C levels and aerobic exercise training.

Mechanism by which aerobic fitness affects neurocognitive health is yet to be clearly elucidated. Despite the evidence showing an association between exercise engagement and improvements in AD biomarkers in cognitively normal older adults [104] and reports of increased aerobic fitness-related increases in brain volume in some studies [105–107], the underlying biological mechanism for these effects needs further clarifications. Given the available evidence, it appears that the effects of exercise on neurocognitive function are mediated through several important pathways. Dyslipidemia, especially low HDL-C levels, inflammation, deranged glucose homeostasis, and endothelia dysfunction are precursors of arteriolosclerosis, decreased cerebral perfusion and cerebral oxygen deprivation, all of which may increase AD risk [108, 109]. Aerobic fitness can increase HDL-C, reduce inflammation [78], improve glucose homeostasis [110], and reduce arteriolosclerosis. Because these benefits can enhance brain perfusion and improve brain oxygenation, likely benefits include reduction in AD risk [111] (Figure 1). Our own analysis of the data from NHANES III supports the advantageous effects of high levels of HDL-C (Figures 3(a) and 3(b)). Because exercise can cause reduction in stress hormone levels known to impair cognitive function [112]; promote neurotrophic changes, nerve cell regeneration, and neurotransmitter repletion, all of which may enhance cognitive performance [113, 114], these effects are likely involved in the mechanism by which aerobic fitness affects neurocognitive function. Since the evidence suggests that exercise can increase solubility of A*β* through increases in HDL-C [62] and favorably regulate hypoxia inducible factor (Figures 1 and 3), these effects may represent alternative important mechanism by which exercise exerts its advantageous effect on neurocognitive function. Training-induced improvements in these putative AD risk factors may precede more distal effects of fitness adaptation such as increased activity in the frontal and parietal regions of the brain and increased gray matter volume in the frontal and superior temporal lobe reported by Colcombe and Kramer, respectively [115, 116]. Collectively, these observations indicate that aerobic fitness may attenuate neurocognitive loss in humans.

## 11. Limitations of Knowledge on the Effects of Exercise on Neurocognitive Function

 While most of the studies on the effects of aerobic fitness on cognition are indicative of its beneficial effects, few limitations of these studies must be pointed out. First, most have not used a standardized exercise protocol, none used randomized controlled design in MCI or mild AD patients. While the evidence supports an overlap of CVD risk with AD risk and the responsiveness of CVD risk factor to fitness adaptation, most of the intervention studies thus far have not explored CVD risk reduction as the mechanism for improvement in cognitive performance. A prospective randomized controlled trial of aerobic fitness with biomarkers and neuroimaging will inform the establishment of causality, and help determine the volume of exercise that is beneficial. Notably, it will lay the groundwork for the determination of the role of genetics in aerobic fitness-related effects and the mechanism by which fitness affects neurocognitive function.

## 12. Apolipoprotein E Gene as a Modifier of AD Risk

### 12.1. APOE Is a Risk Factor for AD

 The evidence suggests that the APOE gene, especially the *ϵ*4 subtype, is a major risk factor for sporadic and late-onset Alzheimer's dementia [117, 118]. There are three known common isoforms of APO (E2, E3, and E4) in humans encoded by the different alleles *ϵ*2, *ϵ*3, and *ϵ*4. It acts as a receptor of ligands, signifying that intraneuronal APOE may be a mechanism by which APOE influences neuronal repair, regeneration, and survival. Further, APOE can interact with *β*-amyloid and tau proteins that are central to the pathogenesis of Alzheimer's dementia. Specifically, the presence of APOE lipoprotein in cerebral blood vessels laden with amyloid *β*-protein (A-*β*) [119] is indicative of the importance of Apoliporotein in the pathogenesis of AD.

### 12.2. APOE Gene May Influence AD Risk through Its Effects on High-Density Lipoprotein Metabolism

Similar to the role of the *ϵ*4 allele APOE gene in the pathogenesis of Alzheimer's dementia, its association with elevated lipid levels [120] and atherosclerosis have also been reported [121]. Genetic variation at the APOE locus can also influence atherogenesis through its effects on HDL-C subfractions. APOE affects the hepatic binding, uptake, and catabolism of several classes of lipoproteins associated with HDL-C subfractions [122, 123]. The *ɛ*2 and *ɛ*4 alleles of the APOE gene are associated with higher and lower HDL-C subfractions, respectively, among different ethnic subgroups and across regional boundaries [124, 125]. Together, these observations suggest that an individuals' genetic makeup, especially at the APOE, locus may interact with the environment to influence HDL-C levels. Because of the importance of apolipoprotein to HDL-C metabolism and its susceptibility to the influence of APOE gene, their combined role in the pathogenesis of AD should be of significant interest.

### 12.3. African Americans: The Role of APO E in AD Risk

Evidence from protein binding indicates that A*β* interacts with APOE in an isoform specific manner, and fibril formation of A*β* is enhanced by the presence of *ɛ*4 allele of the APOE gene. In its physiologic state, APOE is normally present in the brain in association with HDL-C-like particles. In view of the important role of the *ɛ*4 allele and its overrepresentation in AAs, a proportionately higher *ɛ*4-associated AD risk in AAs would be expected. However, some evidence suggests the converse.

The interaction of HDL-C with APOE provides a useful insight into the reduced *ɛ*4 allele-associated AD risk, and the slower rate of AD progression in AAs. Consistently, higher levels of HDL-C have been shown in AAs than elderly Caucasians [126, 127]. In the presence of HDL-C particle, Olesen and colleagues found no direct effect of APOE on amyloid formation [59]. This suggest that, though *ɛ*4 may increase the spontaneous amyloid formation of A*β*, HDL-C-bound A*β* appear to decrease amyloid formation as a result of strong amyloid inhibitory effect of HDL-C. Alternatively, *ɛ*4 allele may influence amyloid formation by affecting the levels of HDL-C-like particles in the brain. Therefore, because AAs have relatively higher levels of HDL-C, it is possible that HDL-C interacts with APOE to reduce the *ɛ*4 allele-related AD risk and, importantly, lower the rates of disease progression in this population.

### 12.4. Combined Effect of APOE Gene and Exercise Training on HDL-C in AAs

 Increased levels of HDL-C and HDL_2_-C are the most significant changes in lipid and lipoprotein levels that occur following aerobic exercise training [128, 129] Results from exercise training studies show higher levels of HDL-C after exercise in most older Whites [130, 131]. Such highly variable responses to a standardized exercise training intervention may implicate genetic factors as contributors.

Across all adult age groups, habitual levels of physical activity are significantly lower in AAs than in Caucasian Americans for both men and women [132, 133]. A sedentary lifestyle among older AAs leads to obesity and higher triglycerides (TG) and LDL-C, but lower HDL-C [134, 135]. Conversely, exercise training can reduce TG and LDL-C and increase HDL-C. Following 10 weeks of aerobic exercise training, Doshi et al. reported an 8% reduction in cholesterol/HDL-C ratio in older AAs, independent of changes in body composition [136]. Conversely, a study in South African Blacks found no significant change in HDL-C levels after exercise training [137]. These studies suggest that exercise training may increase HDL-C levels in some AAs. Significant interactions with APOE genotype are one possible mechanism by which this can occur. Interestingly, our own standardized aerobic exercise training data showed fitness-related increases in the levels of HDL-C particle size and concentration in *ɛ*2/3 and *ɛ*4 AAs, though to a lower extent in *ɛ*4 carriers. Therefore, APOE and other genetic markers may account for some of the disagreements among studies.

In our currently ongoing pilot study, we will collect prospective data on APOE, HDL-C (particle size and concentration), other biomarkers, neurocognitive function, and neuroimaging. Data on the interactive effects of HDL-C, APOE, and aerobic exercise training on neurocognitive function, will be used to inform the power calculation for a full-scale clinical trial to determine the mechanism by which aerobic-fitness affects neurocognitive function.

### 12.5. Summary of Current Knowledge, Gaps

The evidence highlights the central role of CVD risk and chronic cerebral oxygen deprivation to neurocognitive health. Importantly, disorders of brain lipid metabolism are associated with all principal pathological features of AD such as synaptic transmission [44], inflammation, amyloid [45], and tau pathology [44]. HDL-C is the predominant lipoprotein in human brain circulation, and its low levels can impair memory [53–55]. Unlike total cholesterol, its brain levels reflect blood level. Low levels of HDL-C is associated with hippocampal atrophy in aged humans [56] and therefore likely to be involved in the effects of lipids on cognitive function. Because HDL-C can also increase the cellular degradation of A*β*, and decrease A*β*-induced neurotoxicity in neural culture, it is likely that HDL-C also plays an important role in the biochemical properties of A*β* amyloid formation, and AD. Aerobic exercise can increase HDL-C, reduce inflammation, improve glucose homeostasis, and enhance cerebral perfusion.

Cross-sectional and few prospective studies in predominantly normal Whites samples suggested that aerobic fitness can enhance cognitive function [96, 102, 116, 125, 138]. The outcome of these studies are hardly definitive, and the mechanism by which fitness adaptation affect cognitive function remains to be fully elucidated. Though we and others have shown that exercise can increase HDL-C (Figure 3), the effect of aerobic fitness-induced changes in HDL-C on preservation of neurocognitive function is yet to be examined. Further, whether these changes correlate with changes in cerebral glucose homeostasis is not known. Future studies must focus not just on CVD risk factors and brain infarcts, but also on its surrogates such as increased vascular resistance and chronic cerebral oxygen insufficiency with or without infarcts as well as decreased oxygenation associated with age-related decline in pulmonary function. The role of HIF in these cascades of events must also be considered.

Consistently, the APOE gene has been shown to influence both HDL-C metabolism and independently AD risk. In view of the susceptibility of HDL-C to aerobic fitness and the importance of APOE gene to HDL metabolism, it is vital to examine the effects of APOE on AD risk and its relationship to aerobic fitness-induced increases in HDL-C. Given potential multiple ways in which exercise may improve cognitive performance and therefore reduce AD risk and the relatively large aerobic fitness-related effect size reported in many studies, clinical trials are needed to determine the effect of aerobic exercise-training on cognitive function in patients with mild AD, notwithstanding the recent NIA consensus statement on general lack of progress.

The demonstration of training-related improvements in neurocognitive function and regional cerebral glucose utilization independent of or interactively with APOE gene would provide momentum for a large-scale clinical trial. A concomitant improvements in HDL-C and inflammatory markers will significantly advance knowledge of the mechanism by which aerobic fitness affects neurocognitive function. A study with the advantage of an experimental design, the use of a control group, and ability to examine the contribution of putative CVD risk factors to AD development and progression is highly desirable. In addition to informing the mechanism by which aerobic fitness can enhance neurocognitive vitality in humans in a subsequent large-scale clinical trial, it will help quantify the effects of aerobic fitness on biomarkers, neurodegeneration, and brain glucose homeostasis. For populations such as AAs with disproportionately higher rates of CVD risk and pathology, a confirmatory large scale trial will validate the role of aerobic fitness as an adjunct treatment to ameliorate the physical, psychological, and economic burden associated with AD at individual levels. In addition to providing evidence leading to a scientific basis for a change in health policy and standard of care, society is also likely benefit from reduction in the economic burden.

## Figures and Tables

**Table 1:** Number of deaths, population, and rate of death per 100,000 with underlying or contributing cause coded as dementia by division and race in persons aged 65 and over: United States 1999–2004.

Division	Race	Death 65y+	Population 65y+	Crude rate 65y+	Age adjusted rate 65y+
New England	Black or African American	1,683	340,854	494	574
	White	77,719	10,875,302	715	633
Middle Atlantic	Black or African American	10,145	3,119,034	325	362
	White	155,700	28,965,773	538	492
East North Central	Black or African American	17,106	2,836,888	603	671
	White	219,113	31,040,245	706	664
West North Central	Black or African American	3,476	493,880	704	757
	White	115,938	14,864,882	780	687
South Atlantic	Black or African American	37,538	5,553,447	676	731
	White	240,812	36,129,123	667	676
East South Central	Black or African American	11,422	1,798,196	635	625
	White	75,592	11,093,302	681	721
West South Central	Black or African American	12,364	2,158,225	573	597
	White	118,490	18,293,681	648	676
Mountain	Black or African American	1,242	232,688	534	688
	White	78,387	12,005,553	653	687
Pacific	Black or African American	8,352	1,283,968	650	725
	White	181,815	25,078,447	725	683

US total	Black or African American	103,328	17,799,544	581	628
	White	1,263,566	188,249,878	671	647
